# Bone Marrow-Derived Inducible Microglia-like Cells Promote Recovery of Chronic Ischemic Stroke Through Modulating Neuroinflammation in Mice

**DOI:** 10.3390/biomedicines13061347

**Published:** 2025-05-30

**Authors:** Bach Ngoc Nguyen, Tomoaki Kitamura, Shuhei Kobashi, Makoto Urushitani, Tomoya Terashima

**Affiliations:** 1 Department of Neurology, Shiga University of Medical Science, Otsu 520–2192, Japan; ngngbach@belle.shiga-med.ac.jp (B.N.N.); skobashi@belle.shiga-med.ac.jp (S.K.); uru@belle.shiga-med.ac.jp (M.U.); 2 Department of Neurosurgery, Shiga University of Medical Science, Otsu 520–2192, Japan; tk3246@belle.shiga-med.ac.jp

**Keywords:** BM-iMG cells, cell therapy, cerebral blood flow, chronic ischemic stroke, microglia, neuroinflammation

## Abstract

**Background:** Chronic ischemic stroke presents a significant challenge in neurology, with limited therapeutic options available for long-term recovery. During cerebral infarction, anti-inflammatory phenotype microglia/macrophages produce anti-inflammatory cytokines and neurotrophic factors that facilitate the process of brain repair. However, obtaining sufficient anti-inflammatory microglia/macrophages from the human central nervous system is challenging. Bone marrow-derived inducible microglia-like cells (BM-iMGs) with an anti-inflammatory microglial phenotype were explored to induce neuroprotective properties. Here, we transplanted BM-iMGs into the brain of middle cerebral artery occlusion (MCAO) model male mice to explore their potential for treating chronic ischemic stroke. **Methods:** Bone marrow-derived mononuclear cells (BM-MNCs) were isolated from green fluorescent protein mice and incubated with granulocyte–macrophage colony-stimulating factor (GM-CSF) and IL-4 to induce BM-iMGs with an anti-inflammatory phenotype. BM-iMGs were transplanted into the brains of mice on day 14 after MCAO, and behavioral tests, histology, cerebral blood flow, and gene expression were evaluated. **Results:** An intracranial injection of BM-iMGs promoted neurobehavioral recovery, reduced neuronal cell loss, suppressed neuroinflammatory astrocytic and microglial responses in the brain, and increased cortical surface cerebral blood flow in MCAO mice. Furthermore, neuroprotective genes were upregulated, whereas proinflammatory genes were downregulated. **Conclusions:** The intracranial injection of BM-iMG cells shows significant potential as a novel therapy for chronic ischemic stroke.

## 1. Introduction

Ischemic stroke, resulting from cerebral artery occlusion, is recognized globally as a major cause of long-term disability, and effective treatments for enhancing functional recovery post-stroke are still inadequately identified [1]. With the aging population on the rise in many developed nations, the prevalence and death rates from stroke are escalating, making it a significant health and economic challenge [2]. Currently, numerous cell therapy clinical trials for chronic ischemic stroke are underway, yet to date, none has yielded satisfactory outcomes [3]. Therefore, the focus in the chronic stage of stroke is on rehabilitation and aftercare, aiming not only to diminish neurological deficits but also to improve daily functionalities and participation, and to prevent or manage long-term consequences such as spasticity, post-stroke depression, or dementia [4]. This situation highlights the critical and immediate demands for effective treatments, particularly in cell therapy.

Microglia, the principal resident immunological macrophage-like cells of the central nervous system, function as scavengers during inflammation and ischemia, activating swiftly upon cerebral ischemia, peaking in activation with increased numbers and heightened activity 2–3 days post-stroke, and persisting for weeks [5,6,7,8]. Microglia are often classified into proinflammatory and anti-inflammatory phenotypes, reflecting their dual role in ischemic injury [8]. Proinflammatory microglia contribute to neuronal damage through the release of inflammatory cytokines, whereas anti-inflammatory microglia promote tissue repair and neuroprotection via factors such as TGF-β1 [8,9,10,11]. Given this functional duality, shifting microglial polarization from a proinflammatory to an anti-inflammatory state has emerged as a potential therapeutic strategy for mitigating post-stroke neuroinflammation [12,13,14]. Noteworthily, significantly elevated proinflammatory markers were observed two weeks post-ischemic stroke, during the sub-acute and chronic phases, without any corresponding change in anti-inflammatory markers [15,16], potentially contributing to secondary brain damage and hindering neuronal recovery process after the stroke [17]. Thus, we hypothesized that modulating the proinflammatory/anti-inflammatory balance, in favor of anti-inflammation, may reduce the detrimental effects of neuroinflammation during chronic ischemic stroke.

Bone marrow-derived inducible microglia-like cells (BM-iMGs) are originally generated by culturing bone marrow-derived mononuclear cells (BM-MNCs) with granulocyte–macrophage colony-stimulating factor (GM-CSF) and IL-4 to induce a neuroprotective microglial phenotype [18]. BM-iMG cells exhibit a potential neuroprotective function by upregulating anti-inflammatory genes and suppressing proinflammatory genes in the spinal cord of the ALS model mice, as demonstrated in a previous study [18]. Therefore, BM-iMGs are expected to show therapeutic effects in ischemic stroke during the chronic stage. This study aims to investigate the therapeutic potential of BM-iMGs by examining their features and effects in the middle cerebral artery occlusion (MCAO) mouse model of ischemic stroke. In this study, we demonstrated that an intracranial injection of BM-iMGs in MCAO mice significantly improved neurobehavioral recovery, enhanced cerebral blood flow, and reduced neuroinflammation in the chronic phase of stroke. These findings suggest that BM-iMGs hold promise as a novel cell-based therapy for promoting brain repair during chronic ischemic stroke.

## 2. Materials and Methods

### 2.1. Animals

Male 8-week-old severe combined immunodeficiency (SCID) mice were purchased from CLEA Japan, Inc. (Tokyo, Japan). C57BL/6-Tg (UBC-green fluorescence protein [GFP]) 30Scha/J (GFP-tg mice) were acquired from the Jackson Laboratory (Bar Harbor, ME, USA) and prepared for the animal experiments. All mice were housed and maintained at the Research Center for Animal Life Science of Shiga University of Medical Science at a room temperature of 23 °C, under a 12 h light/dark cycle (lights on and off at 8:00 a.m. and 8:00 p.m.). All animals had free access to food and water. A total of 57 mice were used in this study. Among them, 27 mice were assigned to the BM-iMG-injected group, 24 to the PBS-injected control group, and 3 to the day 14 group. Of these, 1 mouse in the BM-iMG group and 2 mice in the control group were excluded for not meeting the inclusion criteria. The remaining mice in each group advanced to the final point of the study.

### 2.2. Animal Model of Middle Cerebral Artery Occlusion (MCAO)

The MCAO procedure was performed according to the method described in a previous study [19,20]. In male SCID mice, a mixed anesthetic including midazolam (4 mg/kg), medetomidine (0.15 mg/kg), and butorphanol (0.2 mg/kg) was intraperitoneally administered to induce anesthesia. The temporal region of the mice was disinfected with 75% ethanol, followed by a linear incision on their scalp. Their temporal muscle was peeled back to expose the skull, and a craniotomy was performed centered over the middle cerebral artery, which can be seen through, to expose the brain. Permanent focal cerebral ischemia was induced by permanently ligating and disconnecting the distal portion of the left middle cerebral artery using bipolar forceps. Immediately after the procedure, the wound was closed, and the mice were allowed to awaken. To verify the effectiveness of the MCAO, neurobehavioral functions were assessed before and after the procedure. From day 1 to day 14, motor and sensory behavioral assessments were conducted on both ipsilateral and contralateral sides to confirm neurobehavioral recovery. The day following surgery, any mice experiencing difficulty in feeding or drinking due to motor dysfunction, or showing excessive surgical sequelae (such as weight loss exceeding 20% of their body weight, inflammation at the wound site, etc.), were euthanized with cervical dislocation as a humanitarian endpoint.

### 2.3. Isolation of Bone Marrow and Induction of BM-iMG Cells from Mice

The BM-iMG cells were prepared using a previously described method [18]. GFP transgenic mice aged 8 to 10 weeks were sacrificed using cervical dislocation, and blood was removed by decapitation. Bone marrow cells were collected from the bilateral humerus, femur, and tibia and then passed through a 70 μm filter and washed. Bone marrow mononuclear cells (BM-MNCs) were obtained with specific gravity centrifugation using 3 mL Ficoll-Paque (GE Healthcare, Chicago, IL, USA), mixed at a 1:1 volume ratio with the suspended bone marrow solution. About 3 × 10^5^ BM-MNCs were cultured in a 35 mm culture dish with 3 mL StemSpan Serum-Free Expansion Medium (STEMCELL Technologies, Vancouver, BC, Canada) in a 37 °C and 5% CO_2_ environment, with the addition of granulocyte–macrophage colony-stimulating factor (GM-CSF) (Wako Pure Chemical Industries, Ltd., Osaka, Japan) and/or IL-4 (Wako Pure Chemical Industries, Ltd.) using the following method: 40 ng/mL GM-CSF for the first 3 days, followed by 40 ng/mL GM-CSF + 40 ng/mL IL-4 for the next 4 days. After 7 days of culture, all these cells were treated with trypsin–ethylenediaminetetraacetic acid (EDTA) (Life Technologies, Carlsbad, CA, USA) for 10 min and then harvested by scraping.

### 2.4. Cytokine Assay

Cell culture supernatant was harvested from both BM-MNCs and BM-iMGs after 7 days of culture as the samples for cytokine assay. For each group, cell culture supernatant from three independent culture dishes were combined to ensure sufficient volume and reduce inter-dish variability. The cytokine analysis was outsourced to GeneticLab (Sapporo, Japan). The samples were analyzed using a multiplex assay, and cytokine concentrations were measured with the Milliplex MAP Kit (HCYTMAG-70K-PX32, Millipore, Burlington, MA, USA) and the Luminex 200 System (Luminex, Austin, TX, USA), employing ELISA technology. The analysis followed the assay protocols and guidelines provided by Millipore. The cytokines measured included G-CSF (granulocyte-colony-stimulating factor), Eotaxin, GM-CSF (granulocyte–macrophage-colony-stimulating factor), IL-3, IL-4, IL-5, IL-7, IL-9, IL-10, IL-12p40, IL-12p70, LIF (leukemia inhibitory factor), IL-13, LIX (LPS-induced CXC chemokine), IL-15, IL-17, IP-10 (interferon-gamma-induced protein-10), KC (keratinocyte-derived chemokine), MCP-1 (monocyte chemoattractant protein-1), MIP-1α (macrophage inflammatory protein-1α), MIP-1β, M-CSF (macrophage-colony-stimulating factor), MIP-2, RANTES, and VEGF (vascular endothelial growth factor). The results were analyzed using the software MasterPlex (version 5.0) (Hitachi Solutions America, Irvine, CA, USA).

### 2.5. Immunocytochemistry Analysis

For immunocytochemistry, BM-iMG cells were cultured in 6-well dishes, fixed with 4% paraformaldehyde, permeabilized with 0.3% Triton X-100, and blocked with 5% normal horse serum. Incubation was performed overnight at 4 °C with a rabbit anti-CD206 antibody (1:500) (Abcam, Waltham, MA, USA), followed by a 1 h incubation at room temperature with an anti-rabbit Alexa Fluor 555 secondary antibody (1:1000). Nuclei were stained with DAPI. Images were taken using a BZ-X800 Keyence (Osaka, Japan) fluorescence microscope. Control excluded the primary antibody to test specificity.

### 2.6. Intracranial Cell Injection of BM-iMG Cells for MCAO Mouse Model

Fourteen days after cerebral infarction, SCID mice were randomly divided into two groups: buffer control and BM-iMG. For the cell injection experiment, the mice were anesthetized by intraperitoneal administration using the mixed anesthetic described earlier. The mice heads were fixed using a stereotaxic instrument (SR-6; Narishige Scientific Instrument Lab, Tokyo, Japan). Their midline scalp was sterilized with 75% ethanol, and then a linear incision was made in the scalp. A small craniotomy was performed on the left side of the cranium, 2 mm posterior from the Bregma point, and 3 mm laterally. Through this burr hole, a 30-gauge needle attached to a Hamilton syringe (Hamilton, Reno, NV, USA) was inserted 2.5 mm depth inside mice’s brains. In the BM-iMG group, BM-iMG cells (1 × 10^6^ cells/3 μL phosphate-buffered saline (PBS)) were injected slowly for 5 min using a stereotaxic microinjector device (IMS-3; Narishige Scientific Instrument Lab, Tokyo, Japan). The buffer control group was similarly injected with PBS. Immediately after these procedures, the scalp was sutured using 5–0 nylon. The mice that showed body weight loss over 20% compared to the time before injection were euthanized immediately in the interest of animal welfare.

### 2.7. Behavioral Tests

The motor and sensory tests were performed pre-operation and on day 1, day 7, day 14, day 15, day 21, and day 28 after stroke using the grid walk test, cylinder test, and von Frey filament test. In the grid walk test, animals were placed on a stainless steel grid floor (20 × 20 cm with a mesh size of 1.0 cm^2^) elevated 20 cm above the floor. The mice performances were recorded for 5 min. As the animals moved across the grid, a foot fault was counted each time a paw slipped through an opening in the grid. The total number of foot faults involving ipsilateral forelimbs per total of 100 steps was counted [21].

For the cylinder test, the mice were placed in a glass cylinder (diameter: 11.5 cm; height: 20 cm), where their movements were recorded. In this test, the number of times the mice reared up and used the forelimbs to make contact with the cylinder wall was measured up to 20 contacts with the cylinder wall or 10 min of recording. In the slow-motion mode, video recordings were evaluated based on the initial use of the forelimb side and fine touches. For fine touches, the number of times the right, left, or both forelimb sides made contact with the wall was counted, and the ratio of use of the affected side was calculated using the following formula: (number of ipsilateral touches + bilateral contacts/2)/(number of ipsilateral + contralateral + bilateral contacts) [22]. The ratio of initial use of the affected forelimb sides was calculated using the following formula: (number of initial uses of ipsilateral side + bilateral contacts/2)/(number of initial uses of ipsilateral + contralateral + bilateral contacts) [23,24].

For the von Frey filament test, mice were placed individually in a transparent cage with a wire mesh floor and allowed to rest for 15 min before the experiment. Mice were then tested with a series of von Frey hair (Tactile Test [Aesthesio] Semmes-Weinstein von Frey Aesthesiometer; Muromachi Kikai Co, Ltd., Tokyo, Japan), providing discrete units of pressure (0.16 g, 0.4 g, 0.6 g, 1.0 g, 1.4 g, 2.0 g, 4.0 g and 6.0 g). The filaments were applied to the mid-plantar surface of the right hind paw until it retracted. Accordingly, using the range of filaments described above, a median weight of 1.0 g was applied first, and the up–down method based on Dixon’s sequential experiments was used to determine the 50% withdrawal threshold [25].

### 2.8. Histological Analysis

Two weeks after injection, MCAO model mice were fixed using transcardial perfusion of 4% paraformaldehyde in 0.1 M PBS. Fixed brain slices were collected and used for immunofluorescence staining. The sections were incubated with 5% normal horse serum in 0.3% Triton X-100 in PBS at room temperature to block non-specific immunoreactions. After blocking, the sections were incubated at 4 °C overnight with following first antibodies: rabbit anti-microtubule-associated protein 2 (MAP2) (1:100) (Cell Signaling Technology, Inc., Danvers, MA, USA), rabbit anti-glial fibrillary acidic protein (GFAP) (1:100) (EnCor Biotechnology Inc., Gainesville, FL, USA), rabbit anti-ionized calcium-binding adapter molecule 1 (Iba1) (1:100) (Wako Pure Chemical Industries, Ltd.), rat anti-CD86 (1:200) (eBioscience, San Diego, CA, USA), and rabbit anti-CD206 (1:100) (Abcam, Waltham, MA, USA). The sections were further incubated at room temperature for 4 h with a secondary antibody donkey anti-rabbit Alexa Fluor 555 (1:1000) (Thermo Fisher Scientific, Waltham, MA, USA) or donkey anti-rat CF568 (1:1000) (Biotium, Fremont, CA, USA), against the corresponding species and mounted with VECSHIELD containing 4′,6-diamidino-2-phenylindole (Vector Laboratories, Inc., Burlingame, CA, USA). Images were captured using a Leica TCS SP8 X confocal laser scanning microscope at 10×, 20×, or 40× objective magnification.

Over three sections of brain per mouse, with an interval of greater than 50 μm, were used to investigate the effect of the treatment on the surrounding tissue starting from the injection site and extending 2 cm caudally. For data analysis, quantification was performed as follows: MAP2 staining intensity was measured with Image J (version 1.53k) in both the ipsilateral and contralateral cortices. The ratio of ipsilateral to contralateral was calculated for each section, and results were plotted for each individual mouse (3 sections per mouse). GFAP-positive and Iba1-positive cells were counted in the ipsilateral thalamus area. The counts from 3 sections per mouse were averaged, and each mouse’s value was plotted in a dot plot format.

### 2.9. Quantitative Polymerase Chain Reaction Analysis

For in vivo gene expression analysis, whole-brain samples from the Bregma point to the Lambda suture were collected on day 14 after injection under deep anesthesia, divided into ipsilateral and contralateral sides, and immediately frozen in liquid nitrogen. Their total RNA was extracted using the NucleoSpin RNA Plus Kit protocol (Takara Bio Inc., Kusatsu, Japan). cDNA was synthesized using reverse transcription with PrimeScript RT Reagent Kit with gDNA Eraser (Takara Bio Inc., Kusatsu, Japan). Quantitative polymerase chain reaction (qPCR) was performed using Luna Universal qPCR Master Mix (M3003L; New England Biolabs, Ipswich, MA, USA) on a LightCycler 480 System II (Roche Diagnostics, Manheim, Germany) according to the manufacturer’s protocol.

The used primer pairs are as follows: glyceraldehyde 3-phosphate dehydrogenase (GAPDH) forward primer, 5′-ATGACCACAGTCCATGCCATC-3ʹ, and GAPDH reverse primer, 5′-GAGCTTCCCGTTCAGCTCTG-3′; CD86 forward primer, 5′-CACGAGCTTTGACAGGAACA-3′, and CD86 reverse primer, 5′-TTAGGTTTCGGGTGACCTTG-3′; CD206 forward primer, 5′-CTATGCAGGCCACTGCTACA-3′, and CD206 reverse primer, 5′-GTTCTCATGGCTTGGCTCTC-3′; inducible nitric oxide synthase (iNOS) forward primer, 5′-ACCCACATCTGGCAGAATGA-3′, and iNOS reverse primer, 5′-AGCCATGACCTTTCGCATTAG-3′; interleukin (IL)-6 forward primer, 5′-ACGGCCTTCCCTACTTCACA-3′, and IL-6 reverse primer, 5′-CATTTCCACGATTTCCCAGA-3′; IL-1β forward primer, 5′-CAACCAACAAGTTGATATTCTCCATG-3′, and IL-1β reverse primer, 5′-GATCCACACTCTCCAGCTGCA-3′; arginase 1 (ARG1) forward primer, 5′-ACCTGCTGGGAAGGAAGAAAAG-3′, and ARG1 reverse primer, 5′-GTTCCGAAGCAAGCCAAGGT-3′; and transforming growth factor-β (TGF-β) forward primer, 5′-CAGAGCTGCGCTTGCAGAG-3′, and TGF-β reverse primer, 5′-GTCAGCAGCCGGTTACCAAG-3′. Quantitative gene expression data were normalized to GAPDH as a housekeeping gene to identify the relative expression levels of the target genes.

### 2.10. Measurement of Cerebral Blood Flow

Cortical surface cerebral blood flow (CBF) in MCAO model mice was assessed utilizing a laser speckle flowmetry imaging system (Omegazone, Omegawave Inc., Tokyo, Japan) as previously detailed [26]. On day 14 after injection, anesthesia was induced and maintained with 2% isoflurane. Mice were placed in the prone position; a vertical midline skin incision (1.0 cm) was made to expose the skull surface to a 780 nm laser light. Scattered light was detected through a hybrid filter using a charge-coupled device camera placed over the mouse’s head. Speckle contrast was analyzed from high-resolution speckle images, indicating high CBF in red and low CBF in blue. Ten consecutive raw speckle images were simultaneously captured and averaged five times. The LIA software (version 4.2) in Omegazone was used for image analysis, with a line drawn from Bregma to Lambda as a guide to the size and location of the regions of interest. On one side of the Bregma–Lambda line, a square was drawn that was divided into four parts. The outer three quarters were the MCA regions, and the caudal halves of the outer quarters were the core regions. The ipsilateral-to-contralateral ratio was calculated using CBF measured in arbitrary units with Omegazone, along with the corresponding area in the contralateral hemisphere.

### 2.11. Statistical Analysis

Data were presented as mean ± standard deviation. The non-parametric Mann–Whitney U-test was used to confirm the differences in medians between the two quantitative groups. Statistical significance was defined by a *p* value of < 0.05. All statistical analyses were performed using SPSS for Windows, version 25.0 (IBM Corp., Armonk, NY, USA).

## 3. Results

### 3.1. Induction of Neuroprotective BM-iMG Cells and Their Therapeutic Application in the Chronic MCAO Mouse Model

**Figure 1:**
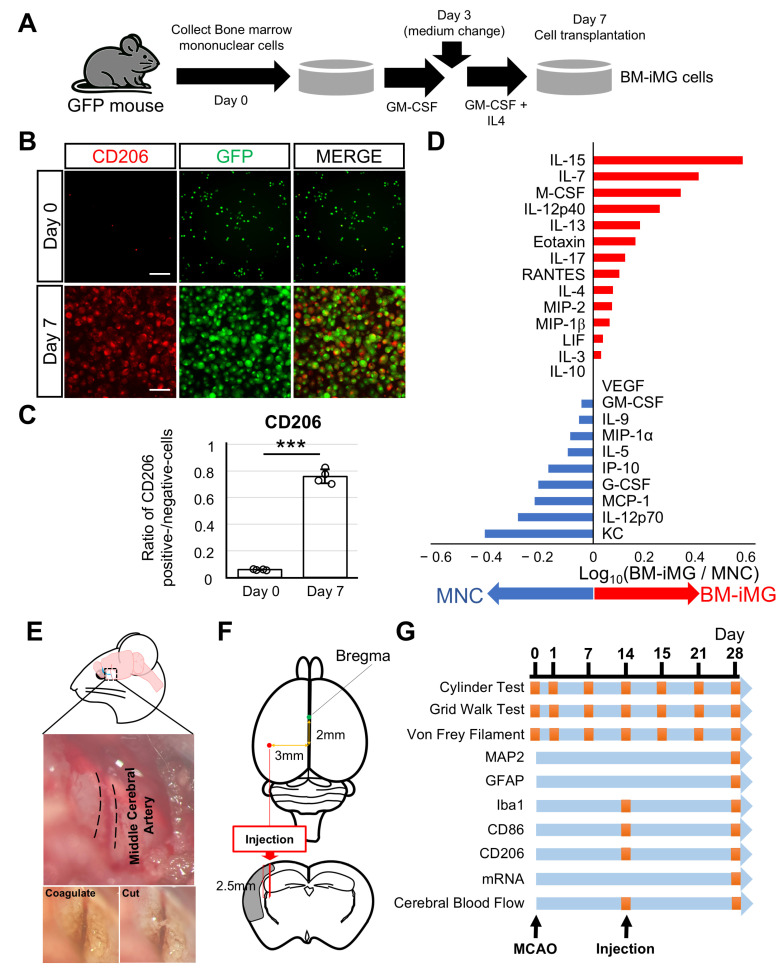
Preparation and features of BM-iMG cells and schematic experimental design for BM-iMG cells injection therapy into a mouse model with chronic cerebral infarction. (**A**) Strategic outlines for the differentiation of BM-MNCs cultured in a regimen that combines GM-CSF and IL-4, encompassing an initial three-day period with GM-CSF alone, followed by a subsequent four-day period of stimulation with both GM-CSF and IL-4. (**B**) Immunofluorescence images of GFP (green) and CD206 bind to Alexa 555 secondary antibody (red) at day 0 and day 7 after culture of BM-MNCs. (**C**) CD206-positive cells ratio is shown for day 0 (*n* = 4) and day 7 (*n* = 4) biologically independent samples. *** *p* < 0.001. (**D**) The ratio of each cytokine concentration in the BM-iMG group against that in the BM-MNC group was logarithmically calculated and shown as red or blue bars. The length of the bars represents the degree of the ratio between the two groups, with red bars indicating the upregulated cytokines in the BM-iMG group and blue bars indicating the upregulated cytokines in the BM-MNC group. (**E**) The schematic diagram of the top indicates the surgical site of the mouse’s head. The middle image displays the left middle cerebral artery (MCA) after left temporal craniotomy in a SCID mouse, with black dotted lines marking the left MCA borders. The pair of bottom images depicts the MCA after electrocoagulation and sectioning. (**F**) Details of the injection point for BM-iMG cells in the brain of MCAO mice. The bregma point is marked by the central green dot, while the injection site is indicated by the red dot on the lower left. BM-iMG cells were intracranially administered on 3 mm left from the midline, 2 mm posterior to the bregma and 2.5 mm deep. (**G**) Outline of the experimental design and assessment procedure. Black arrows indicate the time of left MCAO operation (day 0) and the BM-iMGs administration (day 14). On the 14th day, mice were divided into two categories: PBS CTL (burr hole followed by PBS injection) and BM-iMGs (burr hole followed by BM-iMGs injection). The small orange squares along the blue lines mark the time points for each behavioral or histological analysis (left).

For preparing BM-iMG cells, BM-MNCs were isolated from the bone marrow of GFP transgenic mice and were induced into BM-iMG with an anti-inflammatory microglial phenotype by GM-CSF and IL-4 stimulation as previously described (Figure 1A). To confirm their features, the cell number was counted during culture and immunostaining with CD206, a marker for neuroprotective microglia (Figure 1B and Supplementary Figure S1A). The number of GFP-positive cells expanded approximately sixfold compared to the initial count (Supplementary Figure S1B), and GFP-positive cells hardly expressed CD206 before incubation with GM-CFS and IL-4; however, most of them co-expressed with CD206 on day 7 (Figure 1B). In quantitative analysis, although less than 5% of cells were positive for CD206 on day 0, the population of CD206-positive cells increased to over 70% by day 7 (Figure 1C). In addition, changes in surface antigen expression were confirmed using flow cytometry before and after a 7-day culture of BM-MNCs (Supplementary Figure S1C,D). CD11b and CD206, markers for pan-macrophage/microglia and neuroprotective microglia, respectively, increased significantly to over 70% by day 7 (Supplementary Figure S1C,D). To assess the purity of these cell populations, the presence of CD45-negative cells, representing the mesenchymal stem cell fraction, was evaluated. The inducible cell fraction contamination was found to be less than 1%. 

The neuroprotective effects of BM-iMGs were assessed by analyzing NSC-34 cell viability with or without BM-iMG-conditioned medium using the WST-8 assay. NOC-18 toxicity experiments showed that replacing the medium with BM-iMG-conditioned medium significantly reduced NOC-18-induced cytotoxicity, whereas the control medium had no effect (Supplementary Figure S1E). These results confirm the neuroprotective microglia-like phenotype of BM-iMGs.

To investigate the features of BM-iMGs, the protein expression profiles of cytokines in BM-MNCs and BM-iMGs were compared using ELISA technology. A total of 24 cytokines and chemokines were measured in both groups (Figure 1D). The analysis revealed that 13 cytokines were upregulated (Figure 1D, red bars), 10 were downregulated (Figure 1D, blue bars), and 1 remained unchanged (Figure 1D) in the BM-iMG group compared to the BM-MNC group. Notably, the anti-inflammatory cytokines IL-13 and IL-4 were increased slightly, while proinflammatory cytokines MIP-1α, IP-10, MCP-1, and IL-12p70 were downregulated in the BM-iMG group compared to BM-MNCs (Figure 1D).

Cerebral infarction was induced by coagulation and disconnection of the distal portion of the left middle cerebral artery in SCID mice at eight weeks old (Figure 1E). BM-iMG cells, derived from GFP mice, were transplanted at 2 mm posteriorly and 3 mm laterally from the Bregma point at a depth of 2.5 mm inside the mice brain at the ipsilateral side (Figure 1F). MCAO mice injected with PBS served as the control group. Neurobehavioral tests including grid walk test, cylinder test, and von Frey filament test were performed from pre-MCAO to 14 days after BM-iMG cell injection (Figure 1G). On day 28, the analysis of histology, gene expression, and cerebral blood flow was analyzed to evaluate therapeutic effects compared to control mice (Figure. 1G).

### 3.2. Effects of Intracranial BM-iMG Cell Injection on Neurobehavioral Function and Brain Volume in Chronic MCAO Mice

**Figure 2:**
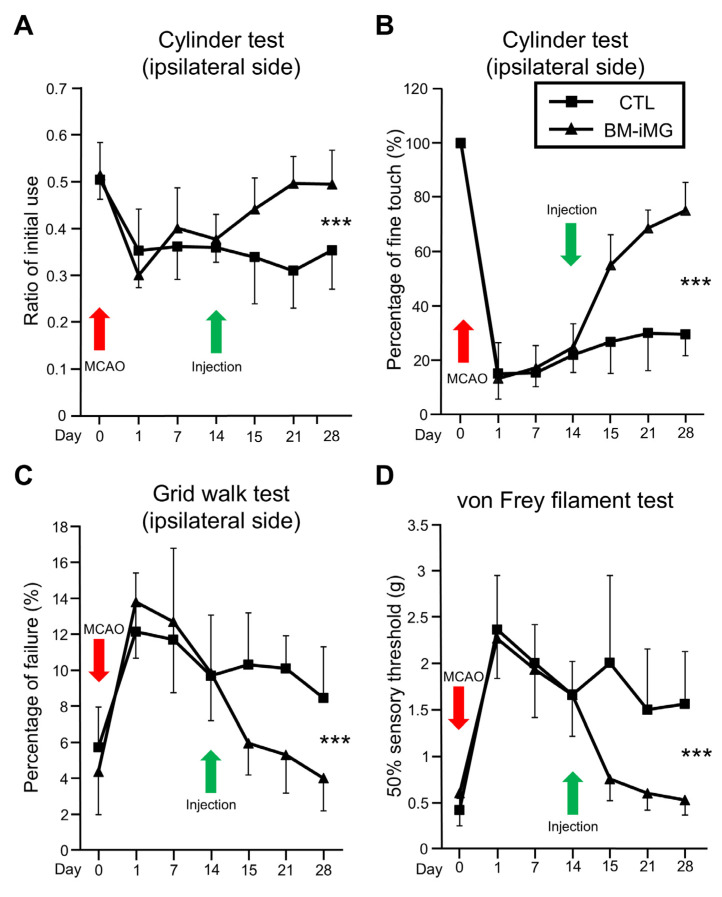
Evaluation of motor and sensory behavior in a mouse model with MCAO procedure (red arrows) following BM-iMG cells injection (green arrows). (**A**,**B**) The use ratio of the affected forelimb during the cylinder test is presented. (**A**) The initial rate of use of the affected forelimb (ipsilateral side) when contacting the cylinder wall, and (**B**) the rate of delicate touch of the affected forelimb (ipsilateral side) upon contacting the cylinder wall, were recorded on days 0, 1, 7, 14, 15, 21, and 28 after the infarction for both PBS control and BM-iMG groups. (**C**) The percentage of failed steps taken with the affected forelimb during all attempts in the grid walk test, is shown, as documented on days 0, 1, 7, 14, 15, 21, and 28 postinfarction for both PBS control and BM-iMG groups. (**D**) The 50% sensory threshold response of the affected hindlimb, as assessed in the von Frey filament test, is shown, recorded on days 0, 1, 7, 14, 15, 21, and 28 after infarction for both PBS control and BM-iMG groups. The PBS control group (*n* = 10) is represented by black squares, and the BM-iMG group (*n* = 11) by black triangles. Error bars indicate the mean ± SD. *** *p* < 0.001; SD denotes standard deviation.

To evaluate the effects of BM-iMG cell injection, cylinder and grid walk tests were performed as motor function before MCAO, and on day 1, day 7, day 14, day 15, day 21, and day 28 after MCAO. In the cylinder test, BM-iMG-treated mice showed a significant improvement in the rate of initial use of the ipsilateral forepaw in comparison with the control group on day 28 after MCAO model induction (Figure 2A). Moreover, the percentage of fine touches on the ipsilateral side decreased similarly in both the BM-iMG and PBS control groups from day 1 to day 14 after MCAO, whereas the percentage of fine touches in the BM-iMG group improved significantly on day 28 compared with that observed in the control group (Figure 2B). For the grid walking test, the frequency of step failure of the ipsilateral forelimb increased to approximately 13% on day 1 after MCAO and then remained at approximately 10% in both groups from day 7 to day 14. However, the administration of BM-iMGs on day 14 significantly reduced the rate of foot faults compared to the control group, which was improved to the same level as before MCAO on day 28 after MCAO (Figure 2C). A von Frey filament test was conducted to assess the sensory function by measuring the 50% withdrawal sensory threshold of the impacted hindlimb. The superficial sensation was impaired on the first day after stroke and spontaneously improved slightly, with a significant recovery effect observed after receiving BM-iMG treatment compared to the PBS control on day 28 after MCAO stroke (Figure 2D).

The stroke-affected area was assessed using Hematoxylin–Eosin (HE) staining after BM-iMG treatment (Supplementary Figure S2). Residual brain areas were visualized through HE staining in pre-treatment (day 14), control, and BM-iMG groups, revealing a consistent lack of cortical area on the ipsilateral side across all groups (Supplementary Figure S2A,B). The residual volume ratio of the entire hemisphere and the cortical area on the ipsilateral side was calculated relative to the contralateral side (Supplementary Figure S2A). The hemisphere residual volume ratios were compared between the BM-iMG and control groups on day 28 in the stroke mice (Supplementary Figure S2C), with no significant differences observed. Similarly, the cortical volume ratios among the three groups showed no significant variation (Supplementary Figure S2A,D). These findings indicate that, while the treatment improved neurobehavioral function, it did not significantly affect the infarction volume on day 28 compared to day 14.

### 3.3. Localization and Characteristics of BM-iMG Cells Post-Injection in the Infarcted Hemisphere of Chronic MCAO Mice

**Figure 3:**
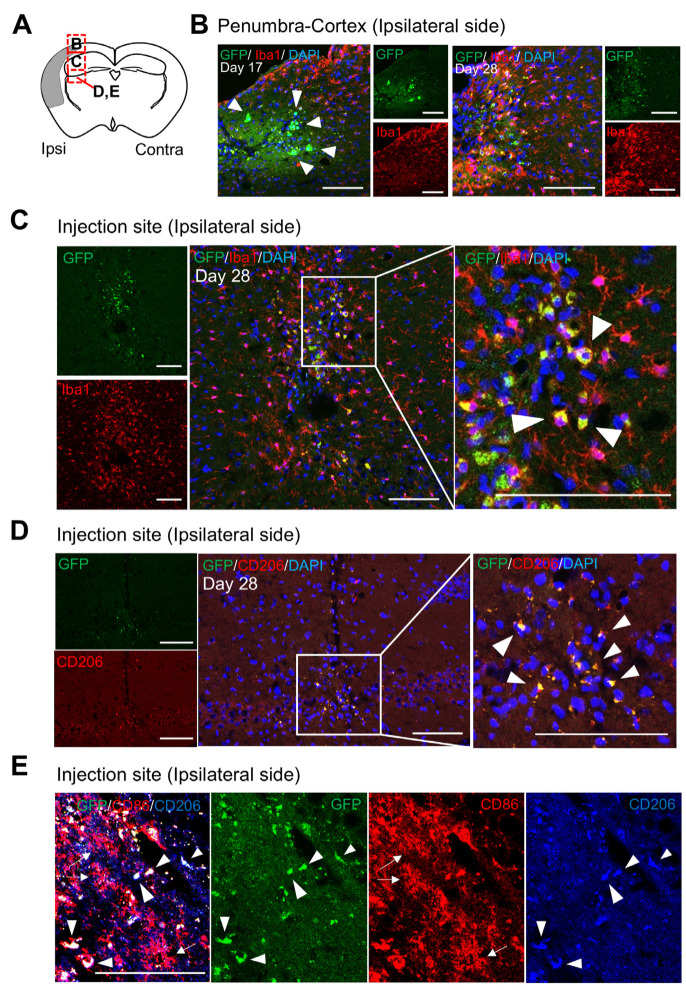
Immunohistochemistry analysis of brains in MCAO mouse model after cell therapy. (**A**) Brain sections were obtained from the injection site of the brain with the infarct lesion on the left side. The dashed red boxes marked (**B**–**E**) in the schematic diagram represent the approximate locations of the following pictures, respectively. (**B**) The panel shows the two series of GFP signal (green), Iba1 immunostaining (red) and DAPI (blue) nuclear staining in the penumbra-cortex area of the brain section 3 days (day 17 in the experimental timeline) and 14 days (day 28 in the experimental timeline) after BM-iMGs injection. Scale bar = 100 μm. (**C**,**D**) Immunohistochemical staining of Iba1 (red), CD206 (red) with DAPI (blue) nuclear stain in the injection area of the brain section 14 days (day 28) after injection of BM-iMGs (green). Each area of the white square in the middle panel is magnified to the right. (**E**) Immunohistochemical staining of CD86 (red), CD206 (blue) with GFP signal in the injection area of the brain section 14 days after injection of BM-iMGs (green). Arrowheads indicate GFP-expressing BM-iMGs in (**B**–**D**). Arrows indicate endogenous cells expressing CD86 in (**E**). Scale bar = 100 μm.

The observed behavioral improvement resulting from BM-iMG cell injection in the MCAO mouse model prompted us to hypothesize that BM-iMG cells might be able to survive injury in vivo. To explore the mechanisms underlying the beneficial effect of BM-iMG cells on behavioral outcomes, immunohistochemistry was performed (Figure 3). Coronal brain sections were obtained from the injection site of BM-iMG cells (Figure 3A, also see Supplementary Figure S3). Sections featuring GFP-positive cells underwent staining with antibodies against Iba1 (microglia/macrophage marker), CD206 (anti-inflammatory microglia/macrophage), MAP2 (neuronal marker), and GFAP (astrocyte marker) to assess the characteristics of the transplanted cells. After 2 weeks, although the GFP signals were attenuated, the transplanted BM-iMG cells could be detected not only in the penumbra-cortex of the injection site (Figure 3B, corresponding to the square of b in Figure 3A) but also in the injection side of the ipsilateral hemisphere (Figure 3C,D, corresponding to the square of c or d in Figure 3A, also see Supplementary Figure S3B,C). Post-BM-iMG cell injection in the ipsilateral injection site, numerous GFP-positive cells co-localized with Iba1 and CD206 but not with CD86, MAP2, or GFAP staining (Figure 3C–E, also see Supplementary Figure S3B,C). These findings suggest that BM-iMG cells were capable of surviving and maintaining their anti-inflammatory microglia phenotype after injection into the affected hemisphere of MCAO mice.

### 3.4. BM-iMG Cells Suppressed Neuronal Cell Loss and Exerted Anti-Inflammatory Effects by Suppressing Neuroinflammatory Astrocytic and Microglial Response

**Figure 4:**
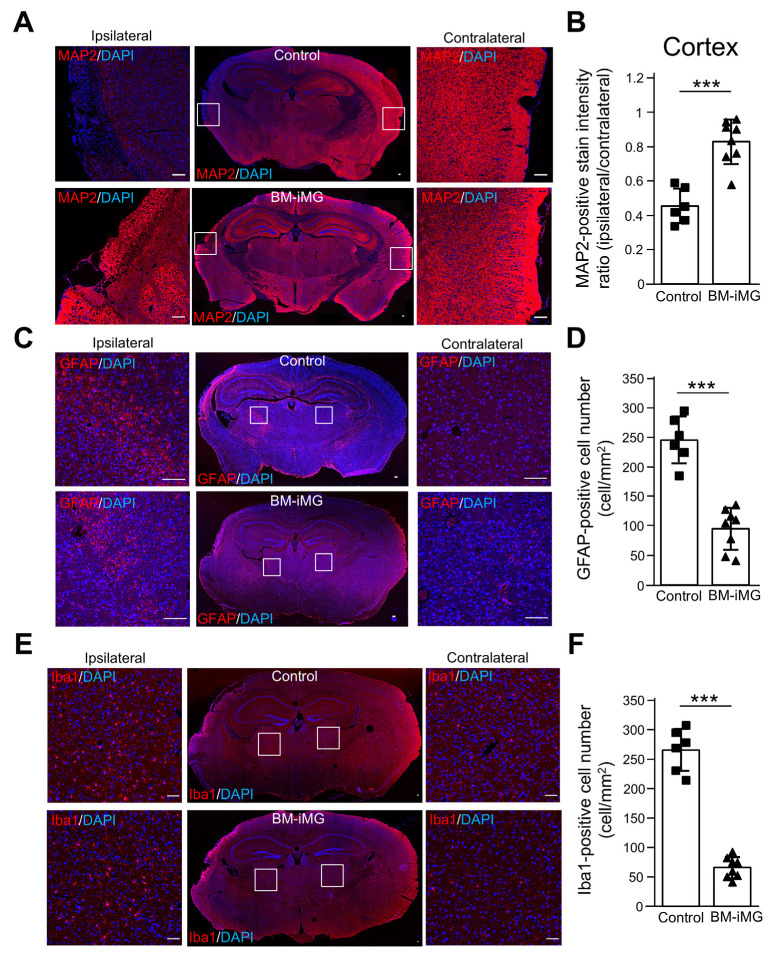
Histological analysis of the infarct area, neuroinflammatory astrocytic and microglial response areas in MCAO mouse brain sections post-cell injection therapy. (**A**) MAP2 immunohistochemical staining (red) in brain sections 28 days after infarction. The upper and lower panels present the PBS control and BM-iMG groups, respectively, with the left and right panels depicting the ipsilateral and contralateral cortex. The central panels display the entire brain section. The white squares (1 × 1 mm) in the central panels are enlarged to either side for detailed viewing. (**B**) The intensity ratio of MAP2 staining between the ipsilateral and contralateral cortex for both PBS control (*n* = 6) and BM-iMG (*n* = 8) groups (3 sections per mouse). Each square (control) or triangle (BM-iMG) represents an individual measurement in a dot plot. Error bars show mean ± SD. *** *p* < 0.001. (**C**) GFAP (red) and DAPI (blue) fluorescent staining in brain sections 28 days post-infarction in the control and BM-iMG groups with the left and right panels depicting the ipsilateral and contralateral cortex. The central panels display the entire brain section. The white squares (500 × 500 μm) in the central panels are enlarged for detailed viewing. (**D**) Quantification of GFAP-positive cells in the ipsilateral thalamus area for both PBS control (*n* = 6) and BM-iMG (*n* = 8) groups (3 sections per mouse). Each square (control) or triangle (BM-iMG) represents an individual measurement in a dot plot. Error bars show mean ± SD. *** *p* < 0.001. (**E**) Iba1 (red) and DAPI (blue) fluorescent staining in brain sections 28 days post-infarction in the control and BM-iMG groups with the left and right panels depicting the ipsilateral and contralateral cortex. The central panels display the entire brain section. The white squares (1 × 1 mm) in the central panels are enlarged for detailed viewing. (**F**) Quantification of Iba1-positive cells in the ipsilateral thalamus area for both PBS control (*n* = 6) and BM-iMG (*n* = 8) groups (3 sections per mouse). Each square (control) or triangle (BM-iMG) represents an individual measurement in a dot plot. Error bars show mean ± SD. *** *p* < 0.001. Scale bars = 100 μm in (**A**,**C**,**E**).

To further evaluate the neuroprotective and anti-inflammatory effects of BM-iMG cells, an immunohistochemistry study focusing on neuronal cell loss and neuroinflammatory astrocytic response was conducted in the MCAO mouse model 2 weeks post-injection. The markers MAP2 and GFAP were utilized to identify neurons and astrocytes, respectively. At day 28 post-MCAO, the cortical lesion on the ipsilateral side exhibited atrophy and reduced MAP2 staining compared to the contralateral side, but this condition was largely reversed by BM-iMG treatment (Figure 4A,B, *** *p* < 0.001). Quantitative analysis in the ipsilateral thalamic area revealed a substantial decrease in the cell number and intensity of the GFAP-positive staining cells in the BM-iMG group compared to the control group at the terminal stage (Figure 4C,D). These results indicate that BM-iMG treatment effectively protects against neuronal cell loss and suppresses neuroinflammatory astrocytic response during the chronic phase following MCAO.

To investigate the mechanism underlying the therapeutic effects of BM-iMG cell administration, double immunofluorescence analysis was performed between GFP-positive cells (transplanted BM-iMG cells) and Iba1, a marker for microglia, in the thalamic region of both BM-iMG transplanted and control groups. Two weeks post-administration, double-positive GFP and Iba1 cells were detected mainly at the injection site, with few detected beyond 0.5 mm from this site; however, Iba1 cell numbers were significantly lower in the BM-iMG group than the control group at the endpoint of the experiment (Figure 4E,F). These results strongly suggest that the injection of BM-iMG cells exerts inhibitory effects on microglial activation, indicating the indirect modulation of microglial activity by BM-iMG cells.

### 3.5. Histochemical Characters of BM-iMG Cells After Injection in MCAO Mice

**Figure 5:**
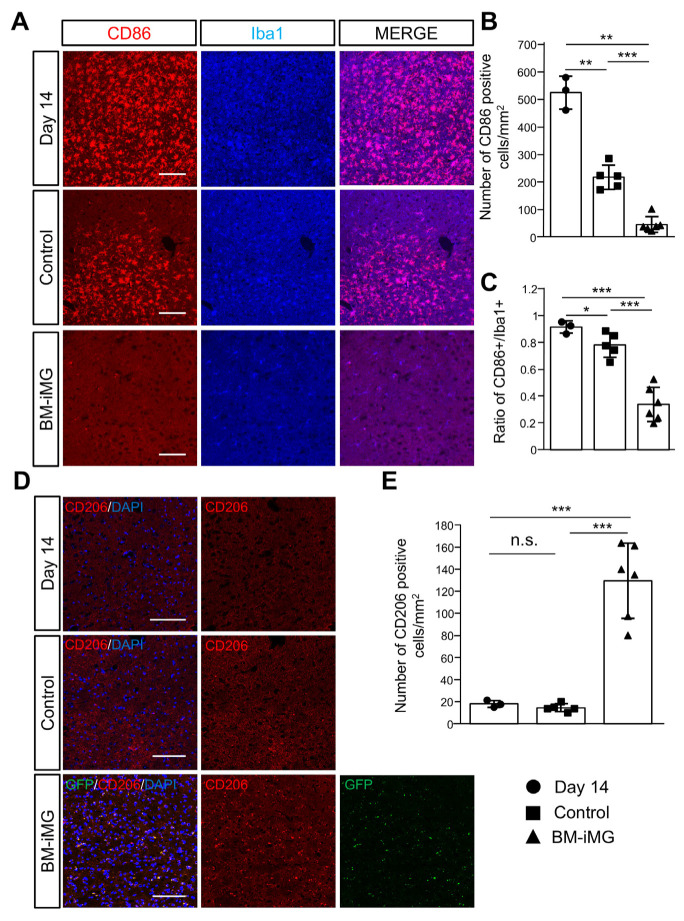
Immunohistochemistry analysis of brains in MCAO mouse model after cell therapy. (**A**) Representative immunostaining of Iba1 (blue) and CD86 (red) in the ipsilateral thalamus area at day 14 after MCAO (upper panel), 14 days after PBS injection for PBS control group (middle panel), and 14 days after BM-iMGs injection for BM-iMG group (lower panel). Scale bars = 100 μm. (**B**,**C**) Quantification of CD86-positive cells (**B**) and ratio between CD86-positive and Iba1-positive cells (**C**) in the ipsilateral thalamus area (500 × 500 μm) for day 14 (*n* = 3) group; PBS control (*n* = 5) group and BM-iMG (*n* = 6) group (totally 3 sections per mouse). Each circle (for the day 14 group), square (for PBS control group) or triangle (for BM-iMG group) represents an individual measurement in a dot plot. Error bars show mean ± SD. * *p* < 0.05, ** *p* < 0.01, *** *p* < 0.001. (**D**) Immunohistochemistry staining of CD206 (red), GFP-expressing BM-iMGs signal (green) and DAPI (blue) in the ipsilateral thalamus area at day 14 after MCAO (upper panel), 14 days after PBS injection for PBS control group (middle panel), and 14 days after BM-iMGs injection for BM-iMG group (lower panel). Scale bars = 100 μm. (**E**) Quantification of CD206-positive cells in the ipsilateral thalamus area (500 × 500 μm) for day 14 (*n* = 3) group; PBS control (*n* = 5) group and BM-iMG (*n* = 6) group (totally 3 sections per mouse). Each circle (for day 14 group), square (for PBS control group) or triangle (for BM-iMG group) represents an individual measurement in a dot plot. Error bars show mean ± SD. n.s.= no significant difference, *** *p* < 0.001.

To explore the therapeutic mechanism of BM-iMG cell injection, we conducted an immunohistochemical analysis to differentiate between proinflammatory and anti-inflammatory microglia in the brains of MCAO mice, before and after administration (Figure 5). Markers CD86 and CD206 were used to identify proinflammatory and anti-inflammatory microglia/macrophages, respectively.

The study found that, in the ipsilateral thalamus area of MCAO brains, CD86-positive (proinflammation) cells were reduced in both control and BM-iMG transplanted groups by day 28, compared to pre-injection at day 14 (Figure 5A). The quantitative evaluation indicated a significant initial presence of CD86-positive cells in this area at day 14, with their numbers diminishing over time in the control group. However, the reduction was more pronounced in the BM-iMG treated group by the end of the observation period (Figure 5B). Furthermore, co-staining for CD86 and Iba1 revealed that the CD86/Iba1 ratio significantly decreased in the BM-iMG treated group by day 28 (Figure 5C). Although no significant change was observed in the number of CD206-positive (anti-inflammation) cells in the control or day 14 groups (Figure 5D,E), there was a significant increase in CD206-positive cells in the BM-iMG treated group, compared to both the control and day 14 groups (Figure 5D,E).

Additionally, double immunofluorescence analysis indicated that a majority of the CD206-positive cells were GFP-positive BM-iMG cells. These findings suggest that BM-iMG cell administration contributes to the reduction in proinflammatory microglia/macrophages during the chronic stage of ischemic stroke, highlighting a potential mechanism behind its therapeutic effects.

### 3.6. Administration of BM-iMG Cells Decreased Proinflammatory and Increased Anti-Inflammatory Gene Expression in Chronic Stage of Ischemic Stroke

**Figure 6:**
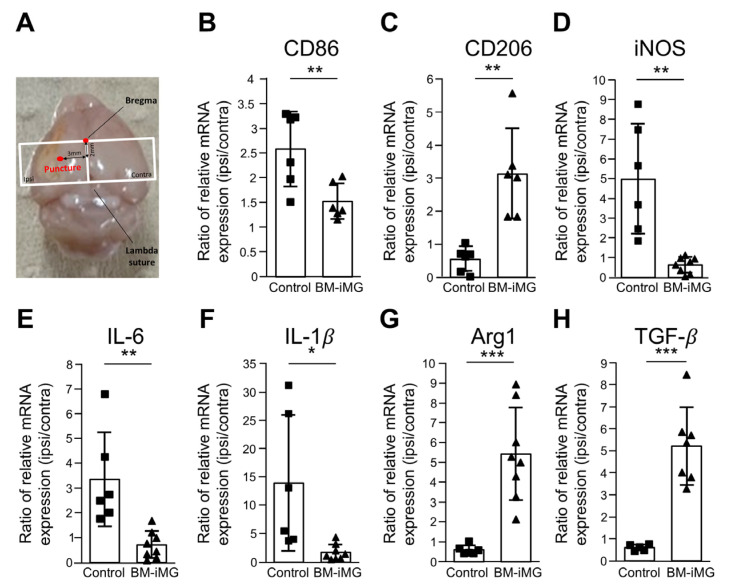
mRNA gene expression analysis in the mouse model of chronic cerebral infarction after intracranial administration of BM-iMG cells. (**A**) Overhead view of the mouse brain displaying cerebral infarction. The central red mark identifies the bregma, while the red spot on the lower left signifies the puncture site, and the white square marks the section removed for mRNA analysis. (**B**–**H**) Relative mRNA expression of (**B**) CD86, (**C**) CD206, (**D**) iNOS, (**E**) IL-6, (**F**) IL1-β, (**G**) ARG1 and (**H**) TGF-β as the ipsilateral to contralateral ratio normalized by GAPDH gene expression in the PBS control (*n* = 6 for CD86, CD206, iNOS, IL-6, IL1-β and ARG1; and *n* = 5 for TGF-β) and BM-iMG (*n*= 6 for CD86 and CD206; *n* = 8 for iNOS, IL-6, IL1-β and ARG1; and *n* = 7 for TGF-β) groups. Each square (for control) or triangle (for BM-iMG) represents an individual measurement in a dot plot. Error bars show mean ± SD. * *p* < 0.05, ** *p* < 0.01, *** *p* < 0.001. ipsi/contra, ipsilateral/contralateral; SD, standard deviation.

Next, we explored whether the injection of BM-iMGs influences microglial polarization by examining the gene expression of proinflammatory markers (CD86) and anti-inflammatory markers (CD206). Gene expression analysis was performed using qPCR on the restricted area of MCAO mouse brain tissues posterior to Bregma, with the puncture site on day 14 after BM-iMG treatment (28 days after cerebral infarction) (Figure 6A). We explored whether the administration of BM-iMG cells influences microglial polarization by examining the gene expression of proinflammatory markers (CD86) and anti-inflammatory markers (CD206). The results showed that the injection of BM-iMG cells into the mice’s brains significantly attenuated CD86 expression and increased CD206 expression of total RNAs in the injection area after MCAO model induction (Figure 6B,C).

As the different phenotypes of microglia are associated with either neurotoxicity or neuroprotective effects, next, we evaluated the therapeutic effects of BM-iMGs on MCAO mouse brains. After 2 weeks post-injection, BM-iMG treatment resulted in the significant downregulation of the proinflammatory genes (iNOS, IL-6, and IL1-β) (Figure 6D–F), concomitant with the upregulation of the expression of neuroprotective genes (ARG1 and TGF-β) (Figure 6G,H) in the MCAO mouse brain in comparison with the brains of the PBS-injected control mice. Collectively, these findings indicated that BM-iMG treatment reduced proinflammatory and increased anti-inflammatory gene expressions in the chronic phase following ischemic stroke, consistent with its neuroprotective function.

### 3.7. Improvement in Cerebral Blood Flow During Ischemic Stroke Recovery by Administration of BM-iMG Cells

**Figure 7:**
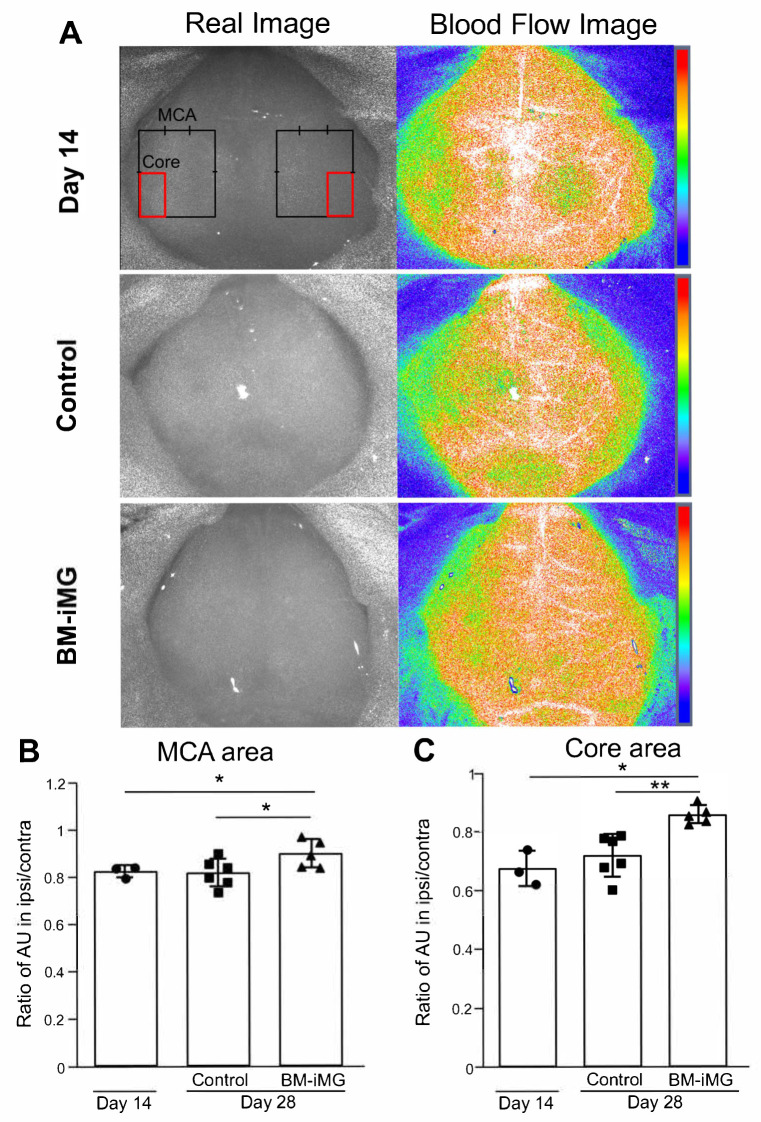
Measurement of CBF in the mouse model of chronic cerebral infarction after injection of BM-iMG cells. (**A**) Panels on the left side show real images from the mouse brain and blood flow images on the right taken using the CBF device 14 days (upper) and 28 days post-infarction in the PBS control (middle) and BM-iMG groups (lower). The heat map images provide a progressive visualization of CBF. (**B**,**C**) The ratio of CBF (ipsi/contra) was presented in AU for day 14 (*n* = 3) group; PBS control (*n* = 6) group and BM-iMG (*n* = 5) group. This is measured in the MCA region (**A**,**B**), indicated by a black solid line (**A**) and the core region (**A**,**C**), represented by a red solid line (**A**). Each circle (for day 14 group), square (for PBS control group) or triangle (for BM-iMG group) represents an individual measurement in a dot plot. Error bars show mean ± SD. * *p* < 0.05, ** *p* < 0.01. AU, arbitrary units; ipsi/contra, ipsilateral/contralateral; SD, standard deviation.

To investigate the potential role of BM-iMGs in CBF improvement during ischemic stroke, cortical surface CBF was measured using a laser speckle flowmetry imaging system, and the ratio between the ipsilateral and contralateral CBF was calculated in the MCA and core infarction area as described in Figure 7A. There was no significant difference in the CBF ratio on day 14 after MCAO induction in comparison with the control group (Figure 7A–C). However, BM-iMG-treated mice displayed significant increases in the CBF ratio in both regions of the MCA and the core of the MCA (Figure 7B,C). Taken together, our data suggest that the CBF in MCAO mice had no significant improvement from day 14 to day 28 during ischemic stroke but was promoted to recovery by BM-iMG cell therapy.

## 4. Discussion

Ischemic stroke remains a major cause of morbidity and mortality worldwide, and the quest for effective therapeutic interventions, especially during the chronic phase, continues to drive research efforts [27]. In this study, we evaluated the therapeutic potential of BM-iMG cells, which exhibit an anti-inflammatory phenotype, in a murine model of chronic ischemic stroke induced by MCAO.

One notable finding was a clear reduction in neuronal cell loss, as observed through immunohistochemistry analysis. The administration of BM-iMG cells demonstrated a significant neuroprotective effect, indicating their potential to mitigate the chronic consequences of ischemic stroke on neuronal integrity. This reduction in neuronal cell loss aligns with the established neuroprotective role attributed to anti-inflammatory microglia, emphasizing their therapeutic potential in the context of ischemic stroke [13,28]. Moreover, in our study, protein profile analysis revealed that, compared to BM-MNCs, BM-iMGs exhibited the upregulation of anti-inflammatory cytokines such as IL-13 and IL-4, while proinflammatory cytokines including MIP-1α, IP-10, MCP-1, and IL-12p70 were downregulated, supporting the anti-inflammatory properties of BM-iMGs. Notably, IL-15 expression was also significantly upregulated in the BM-iMG group, which is of particular interest given its reported role in supporting neural stem cell survival, proliferation, and differentiation, as well as its contribution to neurogenesis and tissue repair following central nervous system injury [29]. These molecular changes highlight the immunomodulatory effects of BM-iMGs. The marked decrease in neuronal cell loss, along with the suppression of proinflammatory microglia/macrophage phenotype following BM-iMGs injection, reflects a strategic redirection of the post-stroke inflammatory environment towards regeneration and repair.

Transcranial MCAO was employed to induce focal cerebral infarction, given its relevance to human cerebral infarction, with more than half of the human cases involving the MCA region [30,31]. This method was chosen among various methods due to its advantages such as the visual confirmation of blood flow blockage, the reproducibility of infarct size and neurological dysfunction, and low mortality rates [32,33]. However, the disadvantages of this surgical procedure include tissue damage, cerebrospinal fluid leakage, and changes in intracranial pressure, temperature, and brain–blood barrier permeability, which were mitigated by establishing a control group for treatment [32,34,35]. SCID mice were selected due to their high reproducibility and long-term survival in modeling cerebral infarction, offering less inter-individual variability in vasculature compared to commonly used C57/BL6 mice [20,36]. Moreover, preclinical research for cell therapy necessitates immunosuppression in mice to prevent xenograft rejection and enables the evaluation of safety and long-term effects [28,37,38,39]. In the normal pathogenesis of stroke, lymphocytes play a complex, “double-edged” role; while some T and B cell subsets exacerbate brain injury through proinflammatory actions, and others may offer neuroprotection, thus complicating the immune response landscape [40]. Utilizing SCID mice, which lack these adaptive immune cells, streamlines the creation of a MCAO model by removing the variable effects of T and B lymphocytes. This approach enhances the evaluation of treatments, such as BM-iMG cell therapy, focusing on the innate mechanisms of brain repair and recovery from stroke, free from the interference of adaptive immune responses.

To our knowledge, this is the first study to demonstrate the mechanism of BM-iMG-mediated immune modulation and neuroprotection in a chronic stroke model. The dynamic interplay between proinflammatory and anti-inflammatory microglia/macrophages following ischemic stroke significantly influences recovery outcomes. In MCAO mice, the expression levels of proinflammatory microglial markers via proinflammatory cytokines (IL-1β, IL-6) and iNOS increased starting at 12 h, peaked by day 14, and then gradually declined and stabilized in the ischemic zone by day 28 post-MCAO [41,42,43]. Conversely, anti-inflammatory markers such as ARG-1 and TGF-β peaked earlier, between days 3 and 5, and returned to baseline by day 14 [44]. This temporal profile informed our decision to transplant cells on day 14, aiming to capitalize on the transition towards anti-inflammatory processes for therapeutic intervention. In our study, we observed the prominent accumulation of proinflammatory microglia/macrophages in the ipsilateral thalamus of control mice, while BM-iMG-treated mice showed the marked suppression of CD86-positive cells and a significant increase in CD206 expression, indicative of anti-inflammatory polarization. These histological findings were supported by mRNA analysis, which confirmed the upregulation of anti-inflammatory gene expression on day 28 in the BM-iMG group. Together, these results highlight the ability of BM-iMG therapy to modulate microglial polarization, supporting a regenerative shift in the post-stroke immune environment. This immune shift may also contribute to the recovery of CBF by promoting vascular stability and reducing inflammation-induced perfusion deficits.

The administration of BM-iMG cells through intravenous or intrathecal routes may offer certain advantages in the treatment of chronic ischemic stroke, although these benefits require further research and validation. Intravenous delivery is less invasive and may allow BM-iMG cells, with their anti-inflammatory properties, to modulate the immune environment systemically. However, this method carries the risk of cells becoming trapped in peripheral organs or failing to effectively cross the blood–brain barrier [45]. In contrast, intrathecal administration is minimally invasive and results in a greater number of cells reaching the ischemic region, significantly reducing damage compared to intravenous delivery in stroke models [46]. Nonetheless, a recent case study reported the risk of inflammatory hypertrophic cauda equina syndrome following intrathecal neural stem cell therapy, highlighting the need for the careful regulatory oversight of intrathecal stem cell administration [47]. Additionally, several clinical trials have demonstrated the safety and improved outcomes of intracranial cell injection during the chronic stage of ischemic stroke [48,49]. These findings support the translational potential of our approach, emphasizing that intracranial cell delivery, although invasive, can be both effective and feasible under certain conditions. Compared to BM-MNCs or mesenchymal stem cells (MSCs), BM-iMG cells specifically engineered to mimic the anti-inflammatory microglia phenotype could offer the more precise modulation of post-stroke inflammation, although their specialization might limit the versatility found in MSCs’ regenerative capacities [50]. Repeated dosages could sustain anti-inflammatory effects, yet the long-term implications of such an approach require further investigation to ensure safety and efficacy.

In this study, we focused on evaluating the efficacy of BM-iMG cells in the chronic stage of stroke using only male mice. This choice was made to maintain consistency and reduce biological variability in the experimental design. Although sex-based differences in stroke outcomes have been documented in humans [51], this study was not designed to assess the impact of sex. Considering the inherent limitations of rodent models and the substantial biological differences between mice and humans, further validation in non-human primates of both sexes will be essential before clinical translation.

## 5. Conclusions

In conclusion, our study provides preliminary evidence supporting the therapeutic potential of BM-iMG cells in chronic ischemic stroke. The observed neuroprotective and anti-inflammatory effects, coupled with the modulation of microglial polarization and increased cortical surface cerebral blood flow, underscore the potential of anti-inflammatory microglia-like cells as a therapeutic intervention. While additional research is needed—such as evaluating long-term safety, optimal delivery routes, dosing regimens, and efficacy in larger animal models—our study suggests that leveraging BM-iMGs could offer a new avenue for treating chronic ischemic stroke and potentially improving outcomes for patients who currently have limited options.

## Data Availability

The data presented in this study are included in the article and Supplementary Materials. Further inquiries should be addressed directly to the corresponding author upon reasonable request.

## Supplementary Materials

The following supporting information can be downloaded at: https://www.mdpi.com/article/10.3390/biomedicines13061347/s1, Figure S1: Differentiation, flow cytometry and quality assessment in evaluating neuroprotective effects of BM-iMGs during cultivation; Figure S2: Hematoxylin-Eosin staining in brain sections in the MCAO mouse model after BM-iMG cells injection; Figure S3: Immunohistochemistry analysis of brains in MCAO mice model after transplantation cell therapy; Supplementary methods.

## Abbreviations

ALS	Amyotrophic Lateral Sclerosis
Arg1	Arginase 1
BM-iMGs	Bone marrow-derived inducible microglia-like cells
BM-MNCs	Bone marrow-derived mononuclear cells
CBF	Cerebral blood flow
EDTA	Ethylenediaminetetraacetic acid
GAPDH	Glyceraldehyde 3-phosphate dehydrogenase
GFAP	Glial fibrillary acidic protein
GFP	Green fluorescence protein
GM-CSF	Granulocyte-macrophage colony-stimulating factor
Iba1	Ionized calcium-binding adapter molecule 1
IL	Interleukin
iNOS	Inducible nitric oxide synthase
MAP2	Microtubule-associated protein 2
MCAO	Middle cerebral artery occlusion
MSCs	Mesenchymal stem cells
PBS	Phosphate-buffered saline
qPCR	Quantitative polymerase chain reaction
SCID	Severe combined immunodeficiency
TGF-β	Transforming growth factor-β
