# Supplementary material for: Bone Marrow-Derived Inducible Microglia-like Cells Promote Recovery of Chronic Ischemic Stroke Through Modulating Neuroinflammation in Mice
Source: Biomedicines. 2025 May 30;13(6):1347. doi: 10.3390/biomedicines13061347 (PMC12189679; doi:10.3390/biomedicines13061347)
Supplement: Supplementary file 1 [file biomedicines-13-01347-s001.zip › biomedicines-3620541-supplementary.pdf]

Supplementary Information

Bone marrow derived inducible microglia like cells promote recovery of chronic ischemic stroke through modulating neuroinflammation in mice

Bach Ngoc Nguyen[1], Tomoaki Kitamura[2], Shuhei Kobashi[1], Makoto Urushitani[1] and Tomoya Terashima[1*]

[1]Department of Neurology, Shiga University of Medical Science, Otsu, JAPAN,

[2]Department of Neurosurgery, Shiga University of Medical Science, Otsu, JAPAN.

Address: Seta Tsukinowa-cho, Otsu,Shiga 520-2192, Japan

***Correspondence:** Tomoya Terashima: tom@belle.shiga-med.ac.jp

Address: Seta Tsukinowa-cho, Otsu, Shiga, 520-2192, Japan.

Supplementary Figure S1

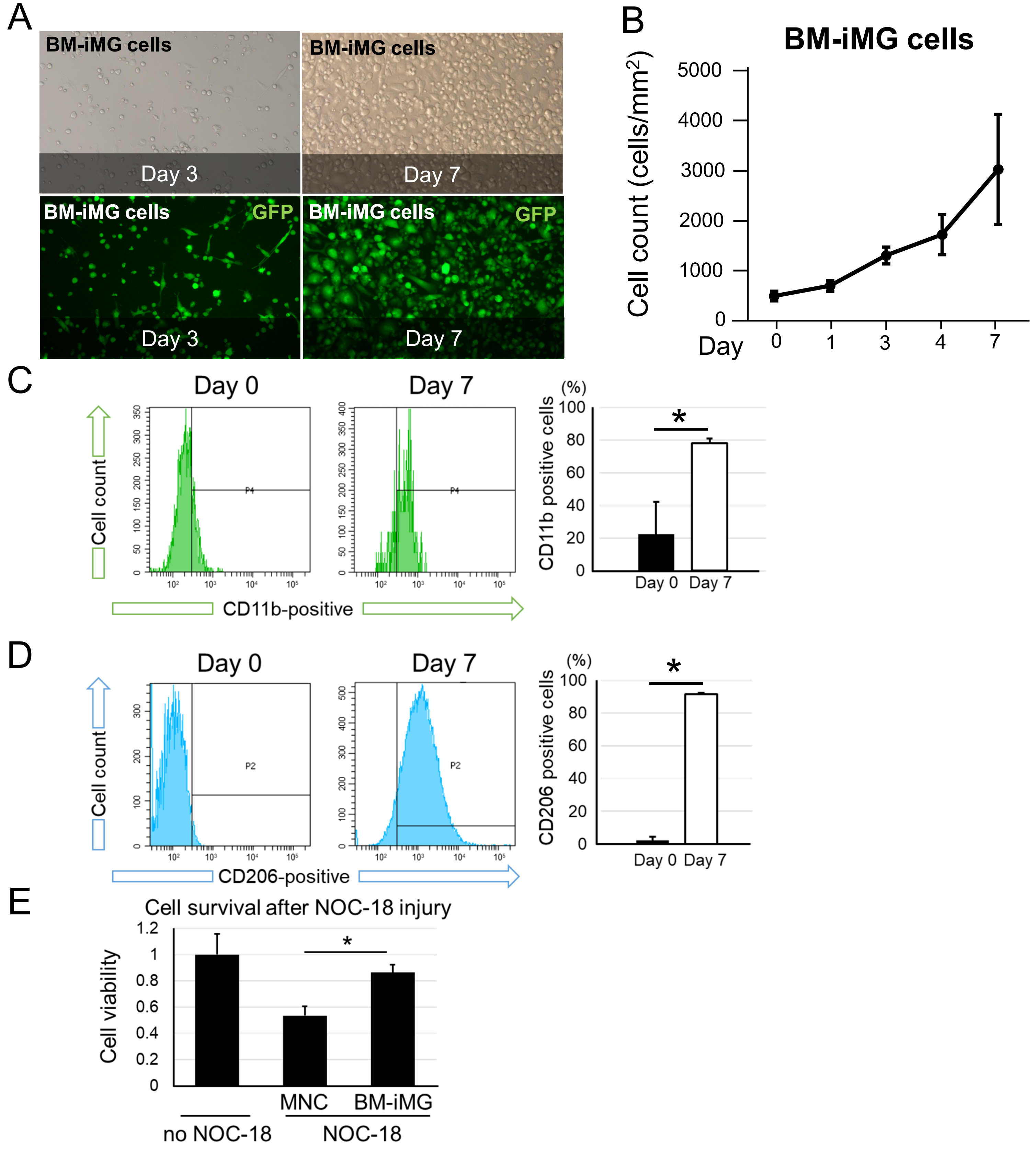

Supplementary Figure S1. Differentiation, flow cytometry and quality assessment in evaluating neuroprotective effects of BM-iMGs during cultivation. **(A)** The upper panels display optical images of BM-iMG cells as seen through an optical microscope. The lower panels exhibit GFP-expressing BM-iMG cells, viewed under a fluorescence microscope. **(B)** The graph shows the quantification of cells during BM-MNCs cultivation (n=4). **(C)** Light green plots display cell counts in CD11b-positive BM-iMGs after incubation, with the black line marking the positive threshold. Percentages for days 0 and 7 were calculated using the right-side population. The bar graph shows CD11b-positive cell ratios on days 0 and 7 (n=4). **(D)** Flow cytometry analysis of CD206 antibody. **(E)** Viability of NCS-34 cells under NOC-18 injury with conditioned medium from MNC or BM-IMG cells culture supernatant. Error bars show mean ± SD. *$P<0.05$. SD, standard deviation.

Supplementary Figure S2

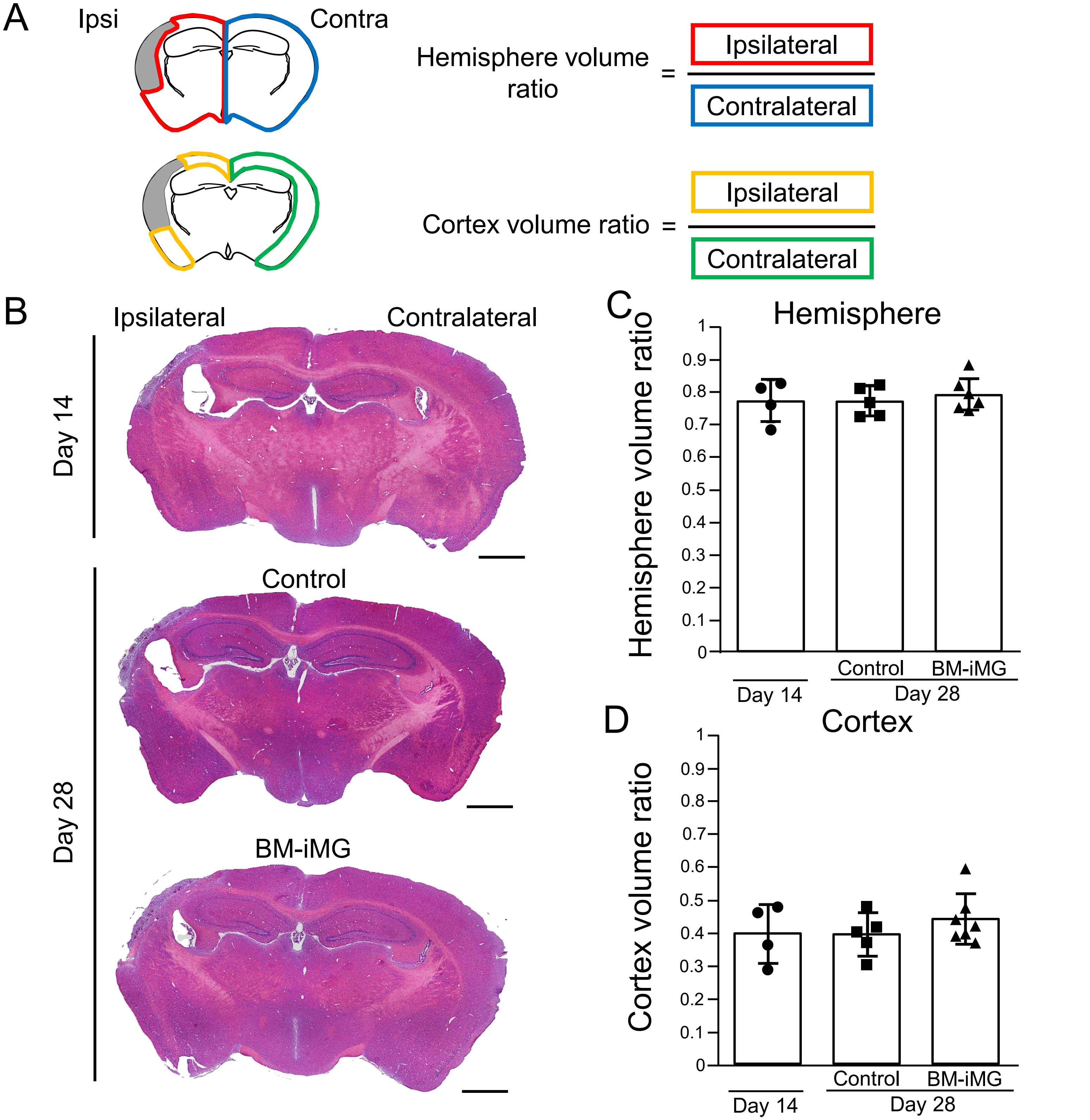

Supplementary Figure S2. Hematoxylin-Eosin staining in brain sections in the MCAO mouse model after BM-iMG cells injection. **(A)** Calculation method of cerebral volume ratio. After HE stain, the whole hemisphere volume ratio was calculated by dividing the whole ipsilateral hemisphere (area surrounded by red line) by the whole contralateral hemisphere (area surrounded by blue line). The volume ratio of cortex was calculated by dividing the ipsilateral cortex remaining (area surrounded by yellow line) by the contralateral cortex (area surrounded by green line). In the schematic illustrations, the cerebral infarction and defect lesion are shown as gray areas. **(B)** Representative HE-stained pictures are shown for day 14 and day 28 in the PBS control and BM-iMG groups after cerebral infarction. **(C,D)** Ipsilateral to contralateral area ratio of the non-infarcted area is shown for day 14 (n = 4) and day 28 in the control (n = 5) and BM-MNC (n = 6) groups in **(C)** hemisphere or **(D)** cortex. Each dot (day 14), square (control) or triangle (BM-iMG) represents an individual measurement in a dot plot. Error bars show mean ± SD. SD, standard deviation.

Supplementary Figure S3

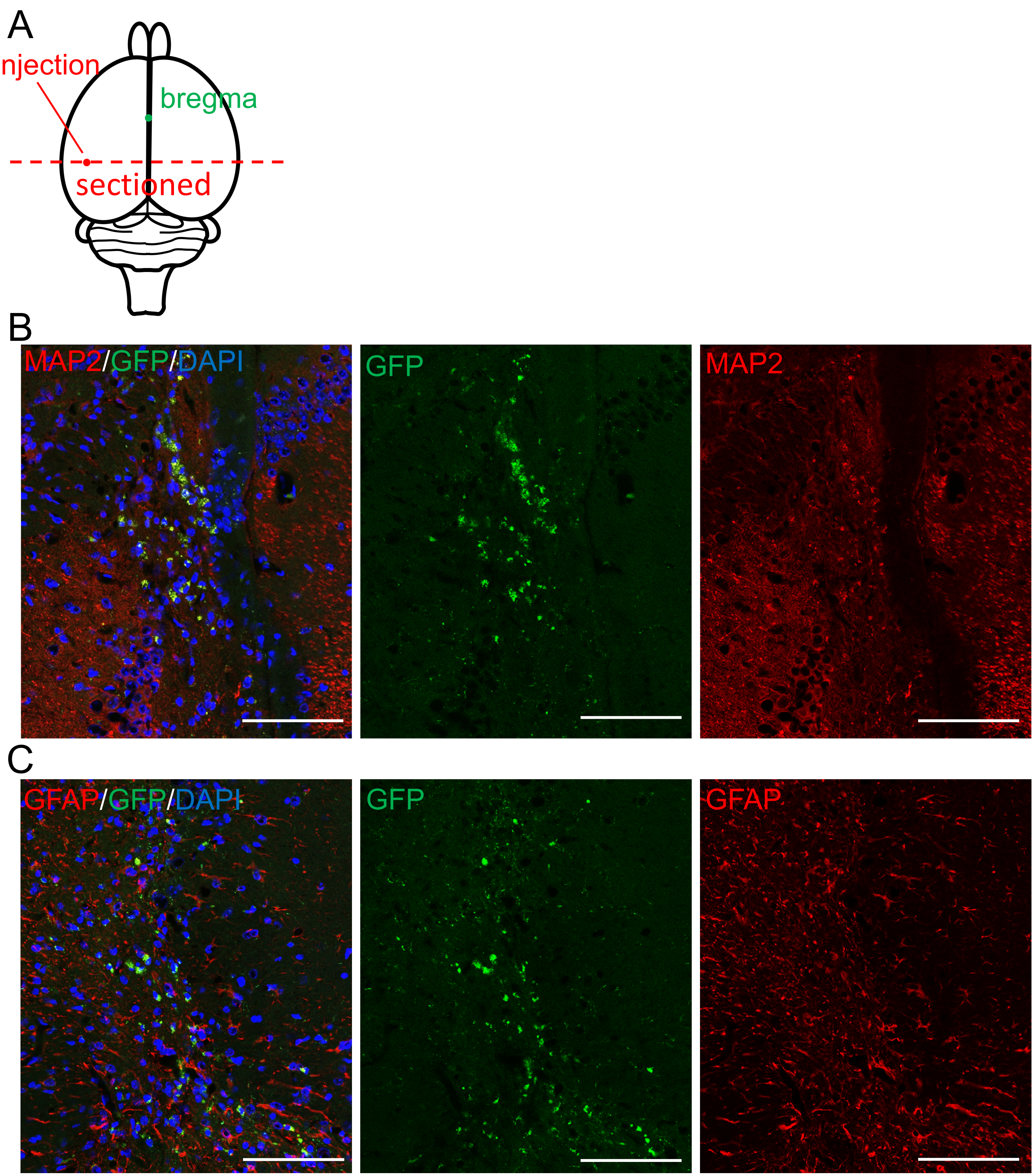

Supplementary Figure S3. Immunohistochemistry analysis of brains in MCAO mice model after transplantation cell therapy. **(A)** Schematic design show mice brain on day 28 after BM-iMG cells transplantation. The central green dot represents the bregma, while the red dot on the lower left marks the puncture point. The red dashed line delineates a horizontal line across the injection site, guiding how the brain was sectioned. **(B,C)** The panels show the two series of GFP signal (green), MAP2 immunostaining (red) **(B)** or GFAP (red) **(C)** and DAPI (blue) nuclear stain in the injection area of the brain section 14 days (day 28 in the experimental timeline) after BM-iMGs transplantation. Scale bar = 100 μm.

Supplementary Methods

Flow cytometry analysis of BM-iMG cells

BM-MNCs derived from GFP mice were cultured with 40 ng/mL GM-CSF for three days, followed by an additional four days with 40 ng/mL GM-CSF and 40 ng/mL IL-4. The cells were treated with trypsin-EDTA for 10 minutes and harvested via scraping. The collected cells were then stained using FITC-conjugated CD11b antibody (BD Biosciences, New Jersey) and Alexa Fluor 647-conjugated CD206 antibody (BioLegend, San Diego, CA). To confirm that BM-iMG cells did not contain mesenchymal stem cells, they were also stained with FITC-conjugated CD45 antibody (BD Biosciences) and analyzed for CD45 positivity. Data acquisition was performed using either a FACS Calibur or FACS Aria flow cytometer (BD Biosciences).

Neuronal cell viability assay

The NSC-34 motor neuron cell line was obtained from CELLutions BIOSYSTEMS (Ontario, Canada). Cells were plated in a 96-well plate at a density of 3×10^4 cells per well using high-glucose DMEM (Wako, Osaka, Japan) supplemented with 10% FBS and allowed to adhere for 3 hours. Once adherence was confirmed, the medium was replaced with high-glucose DMEM containing 40% conditioned medium derived from MNC or BM-iMG cell culture supernatants. To induce neuronal cell death, 1-Hydroxy-2-oxo-3,3-bis(2-aminoethyl)-1-triazene (NOC-18, Dojindo, Kumamoto, Japan) was added at a concentration of 400 µM, and cells were incubated at 37 ° C in a CO_2 incubator for 48 hours. Afterward, the medium was replaced with high-glucose DMEM containing 10% FBS. The survival rate of NSC-34 cells was assessed using the Cell Counting Kit-8 (Dojindo, Kumamoto, Japan), following the manufacturer's instructions. This kit utilizes WST-8, which is reduced by dehydrogenase in viable cells to form WST-8 formazan, producing an orange color. Absorbance of the orange color was measured at 450 nm using a multi-well plate reader (Infinite F200, TECAN, Kawasaki, Japan). The absorbance ratio for each group was normalized to the control group after 48 hours of treatment.

Hematoxylin-Eosin Staining

Hematoxylin and Eosin (HE) staining was performed on frozen brain sections from MCAO mouse brains to visualize tissue morphology. Sections were fixed with cold 4% paraformaldehyde, stained with hematoxylin for 2–5 minutes to highlight nuclei, and counterstained with eosin for 30 seconds to 1 minute to color cytoplasmic and extracellular components. Following staining, sections were dehydrated in graded ethanol solutions, cleared in xylene, and mounted with a permanent mounting medium.
